# Survey of Fleas and Ticks for *Rickettsia rickettsii* and *Rickettsia typhi* in the El Paso Community and Other Areas in Texas, New Mexico, and Ciudad Juarez, Mexico

**DOI:** 10.4269/ajtmh.24-0709

**Published:** 2025-06-17

**Authors:** Karen R. Valdez, Nicole L. Mendell, Angélica María Escárcega-Ávila, Antonio de la Mora-Covarrubias, Florinda Jiménez-Vega, Kenneth A. Waldrup, Veronica Suarez, John C. Morrill, Caroline T. Weldon, Donald H. Bouyer, David H. Walker, Scott C. Weaver, Lucas S. Blanton, Pedro M. Palermo, Douglas M. Watts

**Affiliations:** ^1^ Oak Ridge Institute for Science and Education Fellow, Rickettsial Zoonoses Branch, Centers for Disease Control and Prevention, Atlanta, Georgia;; ^2^ Department of Pathology, University of Texas Medical Branch, Galveston, Texas;; ^3^ Department of Veterinary Science, Institute of Biomedical Science, Autonomous University of Ciudad Juárez, Chihuahua, Mexico;; ^4^ Department of Biological Science, Autonomous University of Ciudad Juárez, Mexico, Chihuahua, Mexico;; ^5^ Department of Biological Science, Institute of Biomedical Science, Autonomous University of Ciudad Juárez, Chihuahua, Mexico;; ^6^ Texas Department of State Health Services, Zoonosis Control, El Paso, Texas;; ^7^ Texas Department of State Health Services, El Paso, Texas;; ^8^ Orion Research and Management Services, Gatesville, Texas;; ^9^ Western Gulf Center of Excellence for Vector-Borne Diseases, Institute for Human Infection and Immunity, University of Texas Medical Branch, Galveston, Texas;; ^10^ Department of Pathology, University of Texas Medical Branch, Galveston, Texas;; ^11^ Department of Microbiology and Immunology and Pathology, University of Texas Medical Branch, Galveston, Texas;; ^12^ Department of Internal Medicine, University of Texas Medical Branch, Galveston, Texas;; ^13^ Department of Biological Sciences, Border Biomedical Research Center, University of Texas at El Paso, El Paso, Texas;; ^14^ University of Texas at El Paso, El Paso, Texas

## Abstract

This survey was conducted with the aim of determining the public health risk of Rocky Mountain spotted fever and murine typhus in the urban and peri-urban areas of El Paso, as well as other areas in Texas, southern New Mexico, and Ciudad Juarez, Mexico. The approach was to assess the diversity of tick and flea species, determine if the ticks and fleas were infected with *Rickettsia rickettsii* and *Rickettsia typhi* (*R. typhi*), respectively, and assess previous human infection with *Rickettsia* species. Ticks and fleas were collected from domestic and wild animals and tested using a nested polymerase chain reaction assay. Human plasma samples were also tested for antibodies using an indirect fluorescence assay. Among 203 fleas, including *Pulex irritans*, *Echidnophaga gallinacea,* and *Ctenocephalides felis* (*C. felis*), collected from wild and domestic small mammals, only one pool of four *C. felis* collected from a dog in the El Paso community was positive for *Rickettsia felis.* All 194 *Rhipicephalus sanguineus* ticks collected from stray and domestic dogs in the El Paso community, southern Doña Ana County, and Ciudad Juarez were negative for *Rickettsia* spp. In Travis County, Texas, a total of 207 ticks collected from white-tailed deer, including 196 *Ixodes scapularis* and 11 *Dermacentor albipictus*, were negative for *Rickettsia* spp. pathogens. Among 375 archived human plasma samples collected in the El Paso community, only two were positive for *R. typhi* antibodies. These preliminary findings suggested that tick- and flea-borne diseases were not a major health risk in the El Paso community or the other areas included in this survey.

## INTRODUCTION

According to the WHO, vector-borne diseases (VBDs) are responsible for more than 17% of all infectious diseases and cause more than 700,000 deaths annually.^1^ In the United States, the number of human cases caused by pathogens transmitted by mosquitoes, fleas, and ticks more than doubled between 2004 and 2016, as indicated by an increase from 27,388 to 47,461 of annually reported cases.^2^ The increasing incidence of tick-borne diseases has been attributed to the emergence of new tick-borne pathogens and an expansion of the geographic ranges of some tick vectors.^3^^,^^4^ Although tick- and mosquito-borne pathogens are the cause of most human disease,^2^ the burden of flea-borne disease is increasing and becoming an important public health concern.^2^^,^^5^ The geographic distribution and host range of fleas have been increasing because of habitat destruction and climate changes.^6^^,^^7^ In the United States, fleas are vectors of *Rickettsia typhi* (*R. typhi*), the cause of murine typhus.^2^ The number of reported annual cases of rickettsioses increased from 1,713 cases in 2004 to 4,269 cases in 2016, thus supporting a growing trend in the disease burden associated with these diseases.^2^

Among tick- and flea-borne diseases, the potential health threats to El Paso, Texas, and the surrounding communities include Rocky Mountain spotted fever (RMSF) and murine typhus. Cases of RMSF have not been reported in the El Paso and surrounding U.S. communities that border Mexico (B. Mayes, personal communication). However, RMSF and murine typhus have been reported in other communities along the U.S.–Mexico border.^8^^–^^13^ As such, the emergence of a *Rickettsia rickettsii* (*R. rickettsii*) tropical lineage maintained in a *Rhipicephalus sanguineus* (*Rh. sanguineus*)–stray dog cycle (subsequently referred to in this report as the brown dog tick cycle) has been the driver of thousands of cases of RMSF and hundreds of deaths during epidemics over the past two decades in impoverished peri-urban and urban communities along the U.S.–Mexico border.^14^^–^^17^ As the other impending threat, the re-emergence of murine typhus has caused cases of febrile illness, as well as severe and fatal disease, with a case fatality rate (CFR) of 1%, primarily recognized in southern California and in the Texas Rio Grande Valley, Texas Gulf Coast, and south central Texas.^12^^,^^13^^,^^18^^–^^22^ Elsewhere along the U.S.–Mexico border, serological evidence of *R. typhi* infection has been detected among humans and domestic dogs in the urban community of Reynosa in the Mexican state of Tamaulipas.^22^

The current ecology and possible disease burden of *R. typhi* and *R. rickettsii* are unknown in El Paso, Texas, and the surrounding communities along the U.S.–Mexico border, as well as other areas in Texas. Therefore, a multifaceted surveillance project was conducted to determine the biodiversity of tick and flea vectors. We also sought to determine if *R. rickettsii* or *R. typhi* could be detected in ticks and fleas collected in peri-urban and urban areas of El Paso (subsequently referred to as the El Paso community), other areas in Texas, southern New Mexico, and Ciudad Juarez, Mexico. Finally, we aimed to determine if humans had been infected with *Rickettsia* species in the El Paso community.

## MATERIALS AND METHODS

### Study site and the collection of ticks and fleas from wild domestic animals.

Ticks were collected from 2019 through 2021 from wild and domestic animals that were live-trapped at selected sites in the El Paso community, Texas, and rural areas in southern Doña Ana County, New Mexico, as well as from stray and domestic dogs in Ciudad Juarez, Mexico. Additionally, a Texas state contractor who controlled the population density of white-tailed deer in prairie and forested land in Travis County, Texas, provided ticks from deer killed by gunning. The ticks were collected under Scientific Permit SPR-0801-168 issued by the Texas Parks and Wildlife Department, Austin, Texas. The fleas were collected from wild animals in peri-urban and urban areas in the Presidio, Fort Hancock, and San Angelo communities of Texas; rural areas in Travis County, Texas; and Anthony City, New Mexico. In addition, the staff of six private veterinary clinics, grooming businesses in the El Paso community, and the El Paso City Animal Shelter Services provided ticks and fleas collected from domestic and wild animals.

All ticks and fleas were collected from wild and domestic animals with forceps and placed in either 15 mL or 50 mL sterile conical tubes. For each collection, the tick, flea, and host animal species were recorded and assigned a number linking the tick and flea samples to the date and location of collection and the host animals.

### Identification and processing of ticks and fleas.

Ticks and fleas were live-transported on ice packs to the University of Texas at El Paso (UTEP) Biosafety Level 2 laboratory and euthanized via exposure to a temperature of –20°C for 2 hours. The ticks and fleas were transferred to a chill table and identified morphologically using a taxonomic key titled *Pictorial Keys: Arthropods, Reptiles, Birds, and Mammals of Public Health Significance*.^23^ Ticks and fleas were disinfected with a 3% bleach solution, washed with a 70% ethanol solution, and then rinsed with sterile deionized water. Fleas were pooled separately (1–7/pool), according to species, date of collection, and host species. The processing of ticks varied depending on the blood-fed status. Briefly, if ticks were not engorged, each one was bisected, and one-half was pooled (according to species, date of collection, and host animal species) and stored at –80°C. Similarly, the other half of each tick was stored at –80°C. If ticks were engorged, four legs were pooled (up to seven ticks per pool), and the other four legs were stored at –80°C. Ticks and fleas were manually homogenized with disposable sterile pestles for 10 minutes in the presence of 200 µL of sterile phosphate-buffered saline (PBS). Tissue suspensions were centrifuged at 3,000 × g for 20 minutes, and the supernatants were stored at –80°C until they were tested for *Rickettsia* species.

### Polymerase chain reaction.

Nucleic acid extracted from fleas and ticks using a Qiagen DNeasy extraction kit (Qiagen, Germantown, MD) was tested for *Rickettsia* species using a nested polymerase chain reaction (PCR) assay.^24^ The quality and quantity of DNA was analyzed using NanoDrop 100 (Thermo Fisher Scientific, Waltham, MA) before attempting pathogen detection. Assays were then performed for the spotted fever group of *Rickettsia* using genus-specific primers with nested PCR of the 17 kilodalton lipoprotein gene, which amplified a 434 base pairs DNA fragment. Reaction mixes for PCR were prepared in 25 µL aliquots utilizing GoTaq green master mix (Promega Corporation, Madison, WI) and 800 µg/ml of bovine serum albumin (BSA). In addition, DNA extracted from a *Rickettsia sibirica* (*R. sibirica*) culture was used as positive control, and DNA extracted from an *R. rickettsii* uninfected Vero cell was used as the negative control. Subsequently, all PCR products were resolved on a 1.5% agarose gel stained with gel red (Biotium, Fremont, CA), rather than ethidium bromide, as used in a previously published study.^24^

### Nucleic acid sequencing.

Tick and flea suspensions with a positive result for rickettsiae on a PCR assay were sequenced to identify the species.^24^ Amplicons were purified by using the Qiagen purification kit (Qiagen) according to the manufacturer’s protocol. Sanger dideoxy sequencing was performed by Genewiz Company, South Plainfield, New Jersey. Sequences were analyzed, aligned, and compared with other sequences available in Genbank (http://www.ncbi.nlm.nih.gov/genbank/).

### Indirect fluorescence antibody assay of human sera for rickettsial antibodies.

Cord blood plasma samples had been collected previously from mothers at the time of delivery during 2015 and 2016 at three hospitals in the El Paso community. The samples were tested for spotted fever group and typhus group antibodies as possible evidence of previous infection by using an indirect immunofluorescence assay (IFA) with a cutoff titer of 1:128 as a presumptive diagnosis, which was performed as described previously.^25^**^,^**^26^ The *R. rickettsii* (Sheila Smith strain) and *R. typhi* (Wilmington strain) antigens were prepared by infecting African green monkey kidney cells (Vero cells; American Type Culture Collection, Manassas, VA). The *R. rickettsii* and *R. typhi* human positive and negative antibody controls were provided by the Rickettsial and Ehrlichial Diseases Research Laboratory at the University of Texas Medical Branch (UTMB), Galveston, Texas. The blocking and wash solution contained PBS with 1% BSA and 0.01% sodium azide. The sample/conjugate diluent contained PBS with 1% BSA and 0.1% Tween 20. In addition to the reagents provided by UTMB, other IFA reagents were obtained from Pan Bio, Inc. (Cypress, CA). A fluorescein-conjugated goat anti-human IgG (KPL, Inc., Gaithersburg, MD) was used to detect IgG antibodies for *R. rickettsii* or *R. typhi* using a fluorescence microscope. For reactive samples, a positive control, a negative control, and positive patient samples were diluted twofold from 1:128 to 1:4,096 and tested to determine endpoint antibody titers.

## RESULTS

A total of 203 fleas (*n* = 50 pools) were collected during the spring, summer, and fall seasons of 2019–2021 from coyotes (*Canis latrans*), raccoons (*Procyon lotor*), gray foxes (*Urocyon cinereoargenteus*), dogs (*Canis lupus familiaris*), and cats (*Felis catus*) in the El Paso community, as well as other areas in Texas and Anthony City, New Mexico (Table 1). The fleas belonged to three genera and three species, including 40 pools of *Pulex irritans* (*P. irritans*; *n* = 156), seven pools of *Echidnophaga gallinacea* (*E. gallinacea*; *n* = 33), and three pools of *Ctenocephalides felis* (*C. felis*; *n* = 14). *Pulex irritans* was the predominant flea species collected, accounting for a total of 156 (76.5%) specimens. They were collected from raccoons (*n* = 9), coyotes (*n* = 70), gray foxes (*n* = 62), dogs (*n* = 11), and cats (*n* = 4). A total of 33 *E. gallinacea* fleas were collected, including 27 from coyotes, two from gray foxes, and four from cats, and 14 *C. felis* fleas were collected from 13 dogs and one raccoon. Among the collection sites, the majority of the fleas (80.3% [163/203]) were collected from animals in the El Paso community, where most of the collection effort was made because of close proximity to the UTEP laboratory. The results include only those animals that were infested with fleas.

**Table 1:** Summary of fleas collected from 2019 to 2021 from domestic and wild animals in central and wouthwestern Texas and Anthony City, New Mexico

Collection Site	*Pulex irritans*	*Ctenocephalides felis*	*Echidnophaga gallinaceae*	Total
Anthony City, New Mexico	4, Dog	1, Dog	0	5
El Paso community, TX	118; Raccoon (*n* = 2), coyote (*n* = 47), gray fox (*n* = 62), dog (*n* = 3), cat (*n* = 4)	12; Dog	33; Coyote (*n* = 27), cat (*n* = 4), gray fox (*n* = 2)	163
Fort Hancock, TX	4, Coyote	0	0	4
Presidio, TX	19, Coyote	0	0	19
San Angelo, TX	4, Dog	0	0	4
Travis County, TX	7, Raccoon	1, Raccoon	0	8
Total fleas	156 (76.8%)	14 (7.0%)	33 (16.3%)	203

Of the 203 (50 pools) fleas that were tested for *Rickettsia* pathogens via PCR, all were negative, except for one pool of four *C. felis* collected in August 2021 from a dog in the El Paso community that tested positive for *Rickettsia felis* (*R. felis*).

Although ticks were collected from the small wild animals that yielded fleas, they were not available for testing. As a result, the ticks that were tested included only those found on dogs and white-tailed deer. These included a total of 401 (*n* = 112 pools) ticks that were collected from 2019 to 2021 in the El Paso community; Travis County, Texas; Doña Ana County, New Mexico; and nearby Ciudad Juarez, Mexico (Table 2). Among these, 194 ticks (*n* = 48 pools) were collected in the El Paso community, Doña Ana County, and Ciudad Juarez. All 194 ticks were collected from dogs, and all were identified as *Rh. sanguineus* (Table 2). In addition, a total of 64 pools consisting of 207 ticks were collected in Travis County from white-tailed deer (*Odocoileus virginianus*). These included 196 *Ixodes scapularis* (*I. scapularis*) and 11 *Dermacentor albipictus* (*D. albipictus*; Table 2).

**Table 2:** Summary of ticks collected from 2019 to 2021 from animals in the El Paso community, as well as other areas in Texas, New Mexico, and Mexico

Collection Sites	*Rhipicephalus sanguineus*	*Ixodes scapularis*	*Dermacentor albipictus*	Total Ticks Collected	Animals
Ciudad Juarez, Mexico	69	0	0	69	Dogs
Doña Ana County, New Mexico	11	0	0	11	Dogs
El Paso community, TX	114	0	0	114	Dogs
Travis County, TX	0	196	11	207	White-tailed deer
Total ticks	194	196	11	401	−

All of the 194 *Rh. sanguineus* (48 pools), including 69 ticks from Ciudad Juarez, Mexico, 11 from Doña Ana County, and 114 from the El Paso community, were negative for *Rickettsia* pathogens (Table 2). Among a total of 64 pools consisting of 196 *I. scapularis* and 11 *D. albipictus* collected from white-tailed deer in Travis County, 10 pools of *I. scapularis* were positive for *Rickettsia buchneri* (*R. buchneri*). The detection of *Rickettsia* spp. in these tick species and the detection of DNA extracted from an *R. sibirica* culture as a positive control confirmed that the PCR assay was performing properly.

A total of 375 cord blood plasma samples collected from mothers at the time of delivery were tested for spotted fever group and typhus group antibodies to determine if humans were being infected by *Rickettsia* in the El Paso community*.* The results indicated that only 0.53% (2/375) were positive for the typhus group antibody, with reciprocal endpoint titers of 1:2,048.

## DISCUSSION

The species of ticks collected from white-tailed deer included 196 *I. scapularis* and 11 *D. albipictus*. All of these ticks were negative for *Rickettsia* pathogens, but 10 pools of *Ixodes scapularis* were positive for *R. buchneri,* an endosymbiont that was originally isolated from the ovaries of *Ixodes scapularis*.^27^ The findings of *I. scapularis* as a common tick species on white-tailed deer was consistent with the results of a study conducted from 2008 to 2019 in South Texas, in which 37 ticks collected from white-tailed deer were all *I. scapularis* and were all positive for a *Rickettsia* endosymbiont.^28^ Overall, our findings provide a preliminary understanding of the biodiversity of tick species parasitizing white-tailed deer in central Texas and demonstrate that these vertebrates are hosts for tick species that are known or implicated as vectors of tickborne pathogens, such as *Anaplasma* spp., Powassan virus, and *Borrelia burgdorferi*.^29^^–^^31^

Among 194 ticks collected from August 2019 through January 2021 around the El Paso community, only the brown dog tick (*Rh. sanguineus)* was identified among those collected from dogs. Whether the taxonomic status of the *Rh. sanguineus* ticks is of the temperate (sensu stricto) or tropical lineage (sensu lato) is unknown; however, they are most likely of the temperate lineage according to a previous report in which seven *Rh. sanguineus* ticks collected in El Paso were identified as being of the temperate lineage.^32^ If they were confirmed as being of the temperate lineage, our observations would differ from those of a survey conducted during 2013 and 2014 in the nearby sister city of Ciudad Juarez, Mexico, which revealed that 99.2% of 1,688 ticks collected from dogs were of the tropical lineage.^33^ Other studies in Mexico revealed that only the tropical lineage of *Rh. sanguineus* was found on dogs.^34^^,^^35^ However, our observation that the brown dog tick was only found on dogs partially differs from the results of a survey conducted in New Mexico, in which *Rh. sanguineus* was collected from mule deer and Rocky Mountain elk, as well as from dogs.^36^ These observations are consistent with the reported findings that the brown dog tick primarily feeds on dogs but may feed on a diverse range of domestic and wild animals, as well as humans.^37^

Our surveillance study, conducted in the El Paso community, other areas in Texas, New Mexico, and Ciudad Juarez, was focused on *R. rickettsii* and *R. typhi* as potential reemerging tick- and flea-borne pathogens.^8^^,^^13^ Of the two diseases, RMSF is the more life threatening, with CFRs ranging from ∼6% to 11.4% in Arizona^14^ and from 14% to 76% during epidemics in northern Mexico.^11^^,^^38^ The epidemics of RMSF have been driven by the brown dog tick cycle in impoverished peri-urban and urban communities in Arizona, as well as in all of the Mexican states along the U.S.–Mexican border, with the exception of the state of Tamaulipas.^8^^,^^11^^,^^39^^,^^40^ As such, the authors of multiple reports have documented the detection of *R. rickettsii* in the tropical lineage of the brown dog tick as the primary vector of *R. rickettsii* during epidemics. As an example, in urban and peri-urban areas of the Mexican community of Ciudad Juarez, which is adjacent to the El Paso community, the prevalence rate of *R. rickettsii* in the tropical lineage of the brown dog tick was 5.0% (3/60) during epidemics of RMSF.^32^ In other U.S.–Mexico border communities, the prevalence rate of *R. rickettsii* in the tropical lineage of the brown dog tick was 3% (2/70) in tribal lands of Arizona;^8^ 0.7% (1/143) to 6.1% (3/49) in Mexicali, Baja California;^41^ and 5.5% (6/109) in the Reynosa community of the Mexican state of Tamaulipas.^42^ However, we did not detect *R. rickettsii* in 194 brown dog ticks collected from dogs in the El Paso community, although the number collected and tested was within the previously reported range to detect *R. rickettsii*. An explanation for our observation that *R. rickettsii* was not detected in the brown dog tick could be the presence of the temperate lineage of the tick in El Paso and the surrounding communities. However, this explanation was not supported by the results of two studies in which *R. rickettsii* was not detected in more than 900 tropical lineage *Rh. sanguineus* collected in Texas and throughout the other U.S. states.^43^^,^^44^

Overall, in addition to not detecting *R. rickettsii* in the brown dog tick, we did not detect antibodies to *R. rickettsii* and other spotted fever group rickettsiae in human plasma samples. As such, this observation is consistent with the historical absence of RMSF in El Paso and the surrounding communities on the U.S. side of the border. Evidence based on the known historic distribution of RMSF in Texas indicates that the area of highest endemicity is in the northeast part of the state. In this area, *R. rickettsii* is transmitted by the American dog tick, *Dermacentor variabilis*, which has never been reported in El Paso, and the known range does not extend west of the Big Bend, Texas.^45^ These observations suggest that *R. rickettsii* is not endemic or enzootic in El Paso and the surrounding communities. However, this observation must not be interpreted as a lack of risk, not only in the El Paso community but also nationally, because of the more recent findings that the tropical lineage of the brown dog tick was not only found in the El Paso community but also throughout the United States.^43^^,^^46^ Therefore, continuous surveillance is warranted because the geographic range of the tropical lineage tick includes regions where *R. rickettsii* is transmitted by *Dermacentor* spp. ticks to humans and dogs. As such, a *R. rickettsii-*infected dog could serve as a source of infection for the tropical lineage of the brown dog tick to initiate a *R. rickettsii–*brown dog tick–dog cycle similar to the driver of the devastating epidemics in eastern Arizona and the northern region of Mexico.^8^^,^^11^

We only collected three species of fleas in the El Paso community and the other areas of Texas. These included the sticktight flea (*E. gallinacea),* the human flea (*P. irritans*), and the cat flea (*C. felis).* Among these three species, the human and sticktight fleas were collected most frequently. The hosts of the *P. irritans* fleas included dogs, raccoons, gray foxes, and cats; for *C. felis*, the hosts included dogs and raccoons; and for *E. gallinacea,* the hosts included cats, coyotes, and gray foxes. The number of cat fleas observed on dogs in our study appeared to be very low because it is usually the leading flea species infesting dogs.^47^ The low number could be attributed to the aridity of El Paso because the distribution of cat fleas has been reported to be limited by low humidity.^48^ Another survey in south Texas detected three flea species, including *Pulex porcinus, Euhoplopsyllus glacialis affinis*, and *E. gallinacea* collected from nilgai, raccoons, cotton-tailed rabbits, coyotes, bobcats, javelina, feral swine, and a black-tailed jackrabbits.^49^ An explanation for the differences in the fleas parasitizing different vertebrate hosts associations in the El Paso community versus southern Texas is unknown, but it is evident that a variety of vertebrate species can serve as hosts for many different flea species. Evidence based on the biology of fleas suggests that the difference is due to the low humidity in the El Paso Trans Pecos ecoregion of Western Texas compared to the high humidity in the Rio Grande Valley of the Rio Grande Plains mammalian ecoregion.^50^

Although *R. typhi* was not detected in our survey, *R. felis* was detected in a pool of four *C. felis* fleas. Additionally, antibodies reactive against typhus group rickettsiae were detected in two of 375 human plasma samples. Our observations are consistent with previous findings in which evidence of murine typhus was not detected in the El Paso community during the 1940s^51^ and 1990s.^52^ Additionally, only one case of murine typhus was reported in 2015 to the Texas Department of State Health Services.^53^ However, these results may also reflect the limited active surveillance in the area or the fact that the cat flea–*R. typhi–*opossum (*Didelphis virginiana*) cycle that is responsible for cases of murine typhus in Texas and California does not exist in the El Paso and surrounding communities.^54^^–^^56^ Opossums are rarely found in West Texas, where they are considered one of the rarest mammals in the region.^57^ The detection of antibodies to *R. typhi* in 0.53% (2/375) of mothers who delivered newborns in hospitals in the El Paso community suggests that murine typhus occurs in this region, albeit at low rates, and may be attributable to the classic rat–rat flea cycle of transmission.

A limitation of our survey was that the scope of the design and methods for collecting ticks and fleas were not appropriate for providing a representative estimate of the diversity of the tick species. As such, it only serves as a preliminary assessment of the potential risk of human disease associated with *R. typhi* and *R. rickettsii* infections in the El Paso and surrounding communities. For such a large sampled geographic area with a relatively populated city (El Paso) at the center, the number of arthropods sampled was relatively small. Additionally, pathogenic rickettsiae are often found to infrequently infect arthropods in nature. For example, in southern California, a few *R. typhi*-infected fleas have been detected in some studies, and in some RMSF endemic areas in the United States, *R. rickettsii* is detected in as few as 0.5% of ticks.^58^^–^^60^ As with other similar studies confined by numerous logistical constraints, sampling was largely performed by convenience, and areas with infected fleas and ticks may have been missed. This was due in part to the failure to collect ticks and fleas using standard collection methods. Another limitation was that the human blood specimens were samples of convenience that likely underestimated the exposure to rickettsiae in the community, which may have been higher in persons with more outdoor exposure, either due to recreational activities or occupation.

The *R. typhi–*cat flea–opossum and dog–brown dog tick–*R. rickettsii* cycles have supplemented the historically identified cycles of these pathogens as a cause of human disease in rural, peri-urban, and urban communities along the U.S.–Mexico border.^8^^,^^12^^,^^21^^,^^22^^,^ The determinants of the distribution of these cycles are unknown. However, our results may reflect the evolving patterns of tick- and flea-borne pathogens. In addition to a regional threat to El Paso and the surrounding communities, these changes could represent impending global health threats. The public health response to these and other VBD threats must include sustained surveillance and the development of effective and innovative prevention and control strategies to mitigate the devastating health impact caused by VBDs.^2^

## CONCLUSION

Although tick- and flea-borne pathogens and diseases are prevalent in the United States and along the border of Mexico, the preliminary findings of this survey suggest they were not a major health risk in the El Paso and surrounding communities, or in the other areas included in this survey. However, the public health response to these and other VBD threats must include sustained surveillance and the development and application of effective and innovative prevention and control strategies to mitigate the potential devastating health impact caused by VBD.
